# Association analysis-based screening strategy for quality markers of Tengdan capsule in the treatment of hypertensive renal disease

**DOI:** 10.3389/fphar.2025.1647921

**Published:** 2025-08-04

**Authors:** Jing Liu, Jun Li, Wei Zhang, Yu Dong, Fang Wang, Jinzhe Liu, Yong Wang, Qian Zhang, Xiaoli Du

**Affiliations:** ^1^ College of Pharmacy, Inner Mongolia Medical University, Hohhot, China; ^2^ Shaanxi Buchang High-tech Pharmaceutical Co., Ltd., Xi’an, China

**Keywords:** Tengdan capsule, hypertensive renal disease, quality marker, transcriptomics, metabolomics

## Abstract

**Background and Aim:**

Hypertensive Renal Disease (HRD) is a chronic and progressive condition driven by sustained high blood pressure, which leads to renal fibrosis and, eventually, end-stage kidney failure. Tengdan Capsule (TDC), a traditional Chinese medicinal formulation, has shown therapeutic potential for managing HRD. However, the specific quality markers (Q-markers) responsible for its efficacy and the underlying molecular mechanisms remain insufficiently understood. This study aimed to identify candidate Q-markers of TDC and elucidate the molecular pathways through which it exerts its renoprotective effects, using an integrative association analysis-based approach.

**Methods:**

The chemical composition of TDC and its potential Q-markers were systematically characterized through *in vitro* and *in vivo* profiling using HPLC-Q-Exactive-MS and chromatographic fingerprinting. Transcriptomic analysis was conducted on angiotensin II-stimulated HEK293T cells to identify differentially expressed genes (DEGs) responsive to candidate Q-markers. Mendelian randomization (MR) analysis based on genome-wide association study (GWAS) data was employed to validate causal genes linked to HRD. Furthermore, untargeted metabolomics was performed to explore metabolic changes associated with transcriptomic targets.

**Results:**

A total of 82 chemical constituents were identified in TDC, 51 detected *in vivo*. Chromatographic fingerprinting across 10 production batches demonstrated high consistency. Integrated analysis of chemical profiling and fingerprint data highlighted Salvianolic acid B (Sal B) as a potential Q-marker. Transcriptomic profiling revealed 210 DEGs enriched in immune and fibrotic pathways. MR analysis identified Killer Cell Lectin Like Receptor D1 (KLRD1) as a protective gene associated with reduced HRD risk (IVW *p* = 0.00797), with no evidence of horizontal pleiotropy or heterogeneity. Metabolomic profiling identified five key metabolites, among which α,α′-diethyl-3,4,4′-stilbenetriol showed a strong correlation with KLRD1 expression, indicating a potential immune–metabolic regulatory axis.

**Conclusion:**

This study presents a novel framework for Q-marker identification in traditional herbal formulations using association-based multi-omics integration. Salvianolic acid B and KLRD1 were key indicators of TDC’s quality and efficacy. These findings offer new mechanistic insights into the action of TDC and support its standardized evaluation and therapeutic application in HRD management.

## 1 Introduction

Hypertensive Renal Disease (HRD) arises from sustained high blood pressure that gradually impairs the renal arterioles. This vascular damage leads to ischemic injury, glomerulosclerosis, and interstitial fibrosis, eventually progressing to end-stage renal disease (Li et al., 2025). Chronic inflammation plays a key role in the development of renal fibrosis. Following injury, renal parenchymal cells release chemokines and cytokines that attract and activate immune cells at the site of damage. Sustained immune activation and the continued release of pro-inflammatory mediators trigger an inflammatory cascade that stimulates fibroblast activation, drives excessive extracellular matrix deposition, and contributes to fibrotic tissue remodeling (Liu D. et al., 2025). These injuries exacerbate the hypertensive state, reinforcing a cycle that makes the relationship between hypertension and kidney damage both biologically intricate and clinically difficult to manage.

Tengdan Capsule (TDC; National Drug Approval No. Z20133012, approved by the China Food and Drug Administration) is an herbal formulation in capsule form, developed by Shanxi Buchang Hi-Tech Pharmaceutical Co., Ltd. Preliminary studies demonstrated that TDC confers renoprotective effects by modulating the TGF-β/Smad signaling pathway, reducing renal fibrosis, and improving kidney function. These findings highlight the therapeutic potential of TDC in the treatment of HRD (Du et al., 2021). However, the effects of TDC on angiotensin II (Ang II)-induced renal injury and inflammation remain inadequately characterized, underscoring the need to elucidate its underlying mechanisms. Advanced analytical techniques such as high-performance liquid chromatography (HPLC) and mass spectrometry (MS) offer powerful means for profiling bioactive compounds within complex herbal formulations (Si et al., 2024; Yu et al., 2025). These techniques enable the identification of bioactive components and support screening potential quality markers (Q-markers), essential for developing quality control systems that correlate chemical profiles with therapeutic effectiveness (Song et al., 2025).

This study systematically characterized the chemical composition of TDC and identified its associated Q-marker through integrated analyses using HPLC-Q-Exactive-MS and chromatographic fingerprinting. Furthermore, an Ang II-induced cellular model of hypertensive renal disease was established to investigate the protective effects of the candidate Q-marker. The findings from this comprehensive approach provide new insights into the active constituents and molecular mechanisms underlying TDC’s therapeutic actions.

## 2 Materials and methods

### 2.1 Drugs and reagents

The TDC batches used in this study (listed in Table 1) were supplied by Shanxi Buchang Pharmaceuticals Co., Ltd. (Shanxi, China). The botanical ingredients *Uncariae Ramulus Cum Uncis*, *Prunellae Spica*, *Suis Fellis Pulvis*, *Taxilli Herba*, *Salviae Miltiorrhizae Radix* et *Rhizoma*, *Astragali Radix*, *Chuanxiong Rhizoma*, *Plantaginis Semen*, *Notoginseng Radix* et *Rhizoma*, and *Stephania tetrandra S. Moore* were authenticated by Professor Qu from Inner Mongolia Medical University (Inner Mongolia, China). Reference standards, including Geniposidic acid (catalog no. DSTDJ004002, purity ≥98%), Salvianolic acid B (Sal B; DSTDD000904, purity ≥98%), Rutin (DSTDL001701, purity ≥99%), Tetrandrine (DSTDF000401, purity ≥98%), and Calycosin-7-glucoside (DSTDM001301, purity ≥98%), were purchased from Chengdu Destar Biotechnology Co., Ltd. (Chengdu, China).

**TABLE 1:** Information on 10 batches of TDC.

No.	Lot no.	No.	Lot no.
S1	210602	S6	210601
S2	210401	S7	210801
S3	210101	S8	210301
S4	211101	S9	210102
S5	210802	S10	210302

All LC-MS grade formic acid, methanol, and phosphoric acid were obtained from Fisher Scientific (Fair Lawn, NJ, United States). Angiotensin II (Ang II; catalog no. PSD241025-768, purity ≥95%) was acquired from Chengdu Pusi Biotechnology Co. (Chengdu, China). Purified water used in the experiments was sourced from Wahaha Group Co., Ltd. (Hangzhou, China).

### 2.2 Preparation of reference solutions

The reference standards Geniposidic acid, Sal B, Rutin, Tetrandrine, and Calycosin-7-glucoside were each dissolved in methanol to prepare stock solutions at concentrations of 324, 215, 212, 251, and 210 μg/mL, respectively.

### 2.3 Preparation of TDC and herbs extracts

Approximately 2 g of TDC was placed in a stoppered conical flask and extracted with 50 mL of 70% ethanol. The mixture was thoroughly shaken and then subjected to ultrasonic extraction for 90 min. After extraction, the solution was centrifuged for 5 min to obtain the supernatant, which was evaporated to dryness. The residue was reconstituted in 10 mL of 15% methanol and filtered through a 0.22 μm membrane to prepare the final test solution (Zhang et al., 2024). Sample solutions of each herb were prepared as described above.

### 2.4 Blood plasma preparation

Sprague-Dawley (SD) rats (n = 12; male; SPF grade; body weight 200 ± 2 g) were obtained from the Animal Center of Inner Mongolia Medical University (Inner Mongolia, China) under certificate number SCXK(m)2020-0003. All animal procedures were approved by the Ethics Committee of Inner Mongolia Medical University (approval no. YKD202301192). Following a 3-day acclimatization, the animals were randomly divided into control and TDC (n = 6 per group). Rats in the TDC group received a suspension of TDC at a dose of 0.91 g/kg by oral gavage, approximating the clinically relevant human equivalent dose (TDC dose = 6 g × 9.1/60 kg). Control rats were administered an equal volume of purified water (10 mL/kg) once daily for three consecutive days. Food and water were withheld for 16 h before the final administration.

Blood samples were collected from the fundus venosus at 0.5, 1.0, 1.5, and 2.0 h after the final dose and transferred into 1.5 mL tubes containing sodium heparin. Plasma was separated by centrifugation at 3,000 r/min for 10 min at 4°C and stored at −20°C. For each time point, 500 μL of plasma (including control) was mixed with 4.5 mL methanol, vortexed for 1 min, centrifuged at 2,000 r/min for 10 min at 4°C, and vacuum-dried. The dried residue was reconstituted in 100 μL methanol, centrifuged again at 12,000 r/min for 10 min, and the resulting supernatant was stored at 4°C in vials with protective liners.

### 2.5 Qualitative analysis

The chemical constituents of TDC were analyzed using a Dionex Ultimate 3000 UPLC system coupled with a Q Exactive quadrupole-orbitrap mass spectrometer (Thermo Fisher Scientific, United States), equipped with a heated electrospray ionization (ESI) source. Chromatographic separation was performed at 40°C using an Agilent ZORBAX SB-Aq column (4.6 × 150 mm, 5 μm). The mobile phase consisted of solvent A (0.1% formic acid in water) and solvent B (methanol), with the following gradient elution program: 5%–5% B (0–5 min), 5%–30% B (5–10 min), 30%–60% B (10–18 min), 60%–80% B (18–25 min), 80%–90% B (25–35 min), and 90%–90% B (35–40 min). The injection volume was 5 μL, and the flow rate was maintained at 0.3 mL/min.

The spray voltages were set at +3.80 kV for positive and −3.20 kV for negative ion modes. The ion transfer tube temperatures were set at 300°C (positive mode) and 400°C (negative mode), while the auxiliary gas heater was maintained at 350°C. Data acquisition was performed in positive and negative ionization modes using full-scan (m/z 110–1200) and data-dependent MS2 (dd-MS2), with a normalized collision energy of 30 eV.

Data acquisition and analysis were performed using Xcalibur 3.0 software (Thermo Fisher Scientific, United States). A detailed in-house database was constructed, incorporating the names, molecular formulas, ionization characteristics, and multistage mass spectral fragmentation patterns of the chemical constituents derived from the herbal components of the TDC decoction (Liu Z. P. et al., 2025; Sun et al., 2024). Unknown chromatographic peaks were interpreted by comparing their primary and secondary mass spectral data with entries from the in-house database and reference compound spectra, enabling the identification of potential chemical constituents.

### 2.6 Establishment of fingerprint

Chromatographic analysis was conducted using a Waters XTERRA MS C18 column (2.1 × 250 mm, 3.5 μm). The mobile phase consisted of solvent A (0.2% phosphoric acid in water) and solvent B (methanol). Gradient elution was applied as follows: 0–12 min, 15%–20% B; 12–24 min, 20%–40% B; 24–45 min, 40%–60% B; 45–53 min, 60%–78% B; and 53–65 min, 78%–95% B. The injection volume was 10 μL, with a 1 mL/min flow rate. UV detection was performed at 254 nm, and the column temperature was maintained at 30°C.

Method validation included evaluations of precision, reproducibility, and sample stability. Precision was assessed by injecting the same sample solution six consecutive times daily. Reproducibility was verified using six independently prepared solutions from the same TDC batch. Sample stability was evaluated by analyzing the same solution at 0, 4, 10, 16, 24, and 30 h post-preparation (Li D. et al., 2024). The similarity among chromatograms of different samples was evaluated using the Similarity Evaluation System for Chromatographic Fingerprint of Traditional Chinese Medicine (Version 2012, Chinese Pharmacopoeia Commission), with correlation coefficients included in the analysis.

### 2.7 Comprehensive analysis of the cellular transcriptome, mendelian randomization (MR) analysis, and metabolome

#### 2.7.1 Cell activity determination of HEK293T cells by CCK8

Human embryonic kidney cells (HEK293T) were obtained from the Cell Bank of the Chinese Academy of Sciences (Shanghai, China). Cells were maintained in DMEM supplemented with 10% fetal bovine serum and penicillin-streptomycin (all reagents from Servicebio, Wuhan, China) at 37°C in a humidified atmosphere containing 5% CO_2_ (Wang C. et al., 2024). The cells were then grown up to ∼80% confluency in 96-well plates, treated with Sal B (2–500 μg/mL) for 24 h, and tested for viability analysis via the CCK-8 assay (Servicebio, Wuhan, China) (Geng et al., 2024; Shang et al., 2024).

#### 2.7.2 Transcriptome mapping and differential expression analysis

HEK293T cells were cultured in 6-well plates until they reached approximately 80% confluence, then divided into three groups: Control, Model, and TDC. Cells in the Model and TDC groups were exposed to angiotensin II (Ang II, 100 nM) for 24 h. The TDC group was further treated with Salvianolic acid B (Sal B, 500 μg/mL) for another 24 h. Total RNA was isolated using the TRIzol reagent kit, following the manufacturer’s instructions. RNA concentration and purity were measured using a NanoDrop 2000 spectrophotometer (Thermo Scientific), while RNA integrity was assessed with an Agilent 2100 Bioanalyzer (Agilent Technologies). Transcriptome sequencing and subsequent analyses were conducted by OE Biotech Co., Ltd. (Shanghai, China).

#### 2.7.3 Mendelian randomization analysis

This study employed genome-wide protein quantitative trait locus (pQTL) data from the Finnish database (https://www.finngen.fi/en), encompassing 2,925 proteins, and integrated it with the previously filtered transcriptomic data described in Section 2.7.2. This approach enabled the refined selection of proteins suitable for Mendelian Randomization (MR) analysis. Summary-level genome-wide association study (GWAS) data for HRD were obtained from the Finnish dataset (finngen_R12_I9_HYPTENSRD), comprising 1367 HRD cases and 345634 healthy controls.

To identify valid instrumental variables (IVs) for assessing HRD risk, single-nucleotide polymorphisms (SNPs) were selected based on the following criteria: a window size ≥10,000 kb, p-value <1 × 10^−5^, and low linkage disequilibrium (R^2^ < 0.001). Weak IVs were excluded using the F-statistic, with an exclusion threshold of F < 10.

Five MR methods were employed to estimate causal relationships: inverse variance weighted (IVW, primary method), MR-Egger, weighted median, simple mode, and weighted mode. Statistical significance was defined as p < 0.05 for the IVW method, with the other methods used for supportive validation. Heterogeneity was assessed using Cochran’s Q test (p > 0.05), and horizontal pleiotropy was evaluated via the MR-Egger intercept test (p > 0.05), both indicating no significant bias. Leave-one-out (LOO) analysis was conducted to confirm the robustness of individual SNP effects.

All MR analyses were performed using R software with the “TwoSampleMR” and “gwasglue” packages.

#### 2.7.4 Determination of mRNA expression by quantitative real-time PCR (qRT-PCR)

Following treatment, total RNA was extracted from cells using TRIzol reagent following the manufacturer’s instructions (Meng et al., 2024) and quantified using a NanoDrop 2000 spectrophotometer. Quantitative real-time PCR (qRT-PCR) was performed on a LightCycler^®^ 480 II Real-Time PCR System (Roche, Switzerland), with GAPDH as the internal control. The primer sequences used are listed in Table 2. Relative gene expression levels were calculated using the 2^−ΔΔCT^ method (Liu Q. et al., 2025; Yuan et al., 2024).

**TABLE 2:** Primer sequences used in mRNA qPCR.

Gene	Forward primer 5′> 3′	Reverse primer 5′> 3′	Product length (bp)
GAPDH	CCT​CAC​AGT​TGC​CAT​GTA​GA	TGG​TAC​ATG​ACA​AGG​TGC​G	69
TGF-β	CAC​CAT​TCA​TGG​CAT​GAA​C	GTG​GAG​CTG​AAG​CAA​TAG​T	123
IL-6	CTT​CTT​CTG​GTC​AGA​AAC​CT	AGT​GTC​CTA​ACG​CTC​ATA​C	85
NGAL	GTG​ACT​ACT​GGA​TCA​GGA​C	GTA​ACT​CGT​TAA​TCC​AGG​GT	92
KLRD1	GGC​AAG​GAA​CTG​AAG​ACT​C	CTA​TAC​AAG​CAA​AGA​AGG​CTC	90

#### 2.7.5 Metabolomics analysis

Cells were resuspended in 1 mL of pre-chilled methanol/water solution (4:1, v/v), transferred into 1.5 mL microcentrifuge tubes, and subjected to sonication in an ice bath (1620 W, 6/4 s on/off cycles) for 30 min. The samples were then precipitated overnight at −40°C. Following precipitation, they were centrifuged at 12,000 rpm for 10 min at 4°C. A 400 μL aliquot of the resulting supernatant was vacuum-dried, reconstituted in 300 μL methanol/water (1:4, v/v) containing 4 μg/mL of internal standard, vortexed, sonicated, and stored again at −40°C overnight. After a final centrifugation at 12,000 rpm for 20 min at 4°C, 150 μL of the supernatant was transferred into LC-MS vials for analysis (Lü et al., 2024).

An ACQUITY UPLC HSS T3 column (100 × 2.1 mm, 1.8 μm) was used for analysis at a column temperature of 45°C. Gradient elution was performed using a mobile phase of 0.1% formic acid in water (solvent A) and acetonitrile (solvent B). The injection volume was 5 μL, with a flow rate of 0.35 mL/min (Liu et al., 2023). Mass spectrometric detection was conducted using an electrospray ionization (ESI) source in both positive and negative ion modes. The instrument settings were as follows: spray voltage, 3 kV; sheath gas, 15 arbitrary units (Arb); auxiliary gas, 5 Arb; and capillary temperature, 320°C.

### 2.8 Statistical analysis

All data are presented as mean ± standard deviation. Statistical comparisons were performed using an unpaired t-test with GraphPad Prism 8.0. A p-value less than 0.05 was considered indicative of statistical significance.

## 3 Results

### 3.1 Identification of 82 constituents in TDC extracts and 51 absorbed prototype compounds in plasma

**FIGURE 1:**
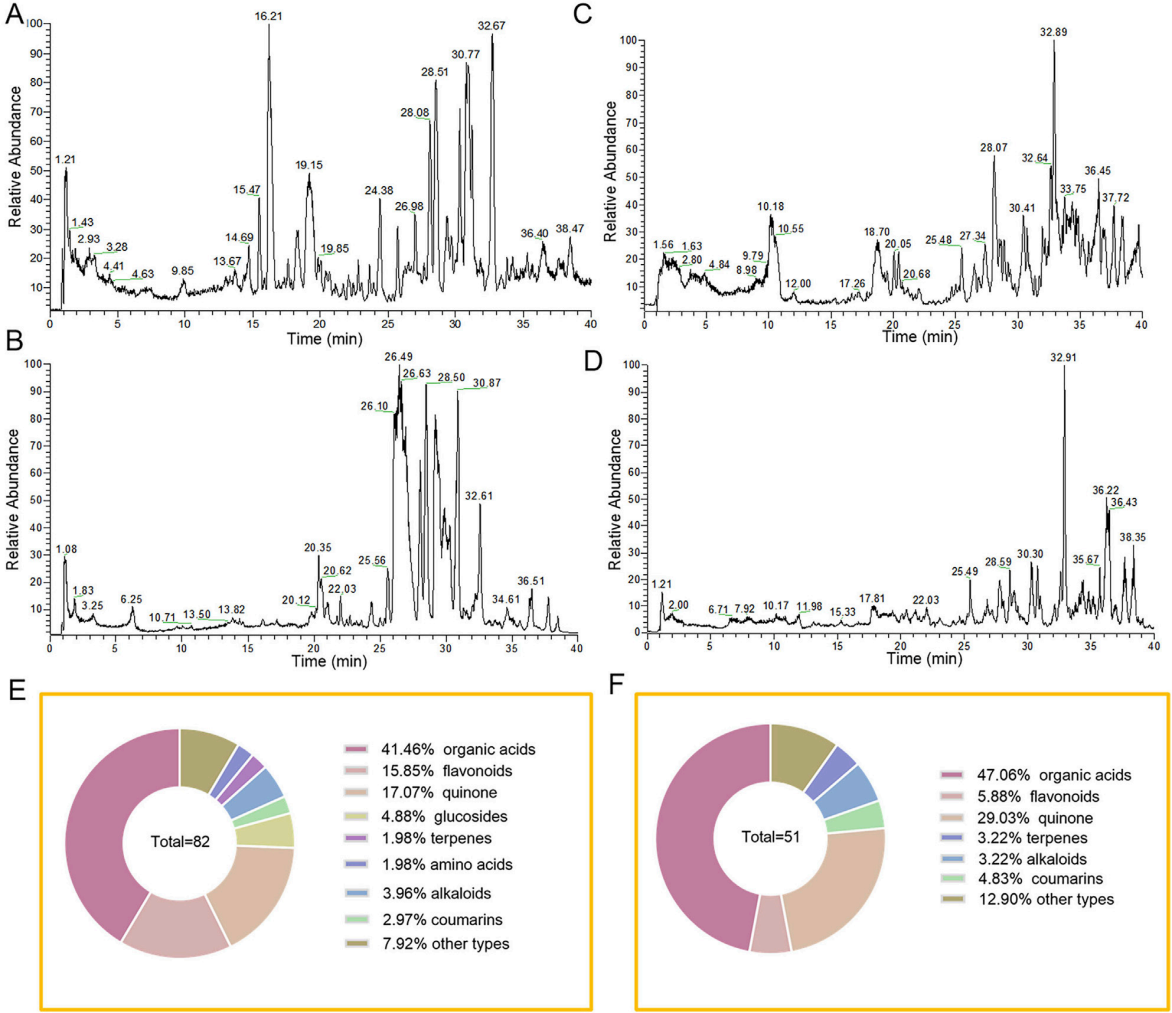
Characterization of chemical constituents of TDC extracts and plasma prototype components by high-resolution mass spectrometry. TDC extracts **(A)** positive ion mode and **(B)** negative ion mode base peak mass spectrum. **(C)** TDC chromatograms of rat plasma analytes in positive and **(D)** negative ion modes. **(E)** TDC extracts the composition. **(F)** TDC plasma prototype components composition.

A total of 82 compounds were identified in TDC based on their mass-to-charge ratios (m/z) and secondary fragmentation patterns (Supplementary Table S1). Analysis using both positive and negative ion modes (Figures 1A,B) classified these compounds into several categories: 13 flavonoids, 2 coumarins, 34 organic acids, 4 glucosides, 2 terpenes, 14 quinones, 4 alkaloids, 2 amino acids, and 7 other compounds (Figure 1E).

Comparative analysis of mass spectra from TDC extracts, blank plasma, and plasma samples post-administration, along with detailed primary and secondary mass spectrometry data for each chromatographic peak, led to the identification of 51 prototype compounds absorbed into the bloodstream (Figures 1C,D; Supplementary Table S2). These included 24 organic acids, 3 flavonoids, 12 quinones, 2 coumarins, 3 alkaloids, 2 terpenes, and 5 compounds of other types (Figure 1F). Several of these constituents were definitively identified by matching retention times and mass spectra with those of reference standards. The absorbed plasma components likely represent the bioactive substances responsible for TDC’s therapeutic effects against HRD.

### 3.2 Evaluation of fingerprint consistency and compound attribution of common peaks in TDC

**FIGURE 2:**
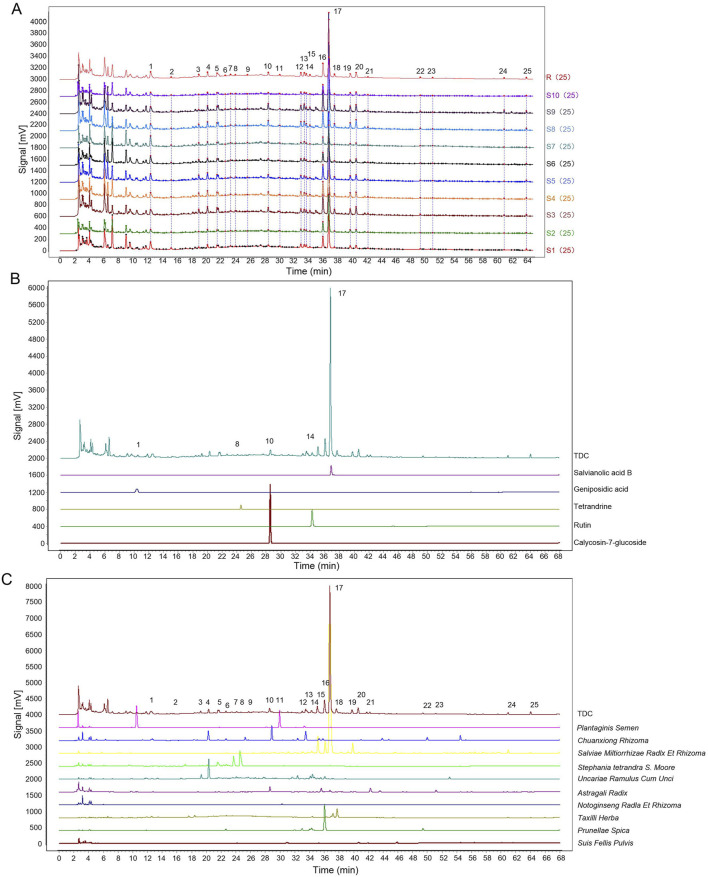
Results of fingerprinting analysis. **(A)** The overlay fingerprint chromatogram of 10 TDC batches. **(B)** Common peaks identified by reference. **(C)** Fingerprint of TDC and its herbs.

The qualitative analysis of TDC’s chemical composition was the foundation for fingerprint development by providing key compositional data. Chromatographic profiling was performed using the Similarity Evaluation System for Chromatographic Fingerprints of Traditional Chinese Medicine (2012 version). Sample S1 was designated as the reference, and a median method with a 0.1 time window width was applied. Following multi-point correction and data alignment, a standard control fingerprint was generated. Overlaid HPLC fingerprints from ten different TDC batches were also produced (Figure 2A). Similarity indices among these batches ranged from 0.902 to 0.998 (Table 3), indicating high batch consistency.

**TABLE 3:** Similarity evaluation results of 10 batches of TDC samples.

Batch	Similarity	Batch	Similarity
S1	0.990	S6	0.972
S2	0.974	S7	0.902
S3	0.998	S8	0.998
S4	0.997	S9	0.996
S5	0.989	S10	0.976

Salvianolic acid B (Sal B, Peak 17) was selected as the internal reference peak, and 25 common fingerprint peaks were identified across the ten batches. Precision testing of the 25 compounds using six replicates showed that the relative standard deviations (RSDs) for stability, precision, and repeatability were below 5%. Reference standards confirmed the identification of Geniposidic acid (Peak 1), Tetrandrine (Peak 9), Calycosin-7-glucoside (Peak 10), Rutin (Peak 14), and Sal B (Peak 17) (Figure 2B).

Fingerprint comparisons between TDC and its herbal components (Figure 2C) revealed 25 distinct chromatographic peaks. Among these, shared peaks were attributed to specific herbs: 4 peaks from *Uncariae Ramulus Cum Unci* (Peaks 3, 4, 14, 16), 4 from *Prunellae Spica* (Peaks 7, 12, 16, 22), 1 from *Suis Fellis Pulvis* (Peak 20), 3 from *Taxilli Herba* (Peaks 18, 24, 25), 4 from *Salviae Miltiorrhizae Radix et Rhizoma* (Peaks 15, 16, 17, 19), 3 from *Astragali Radix* (Peaks 10, 21, 23), 4 from *Chuanxiong Rhizoma* (Peaks 2, 4, 6, 13), 2 from *Plantaginis Semen* (Peaks 1, 11), 1 from *Notoginseng Radix et Rhizoma* (Peak 11), and 3 from *Stephania tetrandra S. Moore* (Peaks 5, 8, 9). The relative contribution of each herb to the fingerprint profile was quantified and is summarized in Table 4.

**TABLE 4:** Contribution of individual herbs to common peaks.

Herb	Number	Rate (%)
*Plantaginis Semen*	5	20
*Chuanxiong Rhizoma*	7	28
*Salviae Miltiorrhizae Radlx Et Rhizoma*	6	24
*Stephania tetrandra S. Moore*	5	20
*Uncariae Ramulus Cum Unci*	8	32
*Astragali Radix*	5	20
*Notoginseng Radla Et Rhizoma*	1	4
*Taxilli Herba*	5	20
*Prunellae Spica*	7	28
*Suis Fellis Pulvis*	1	4

### 3.3 Screening and identification of Sal B as a quality marker of TDC

**FIGURE 3:**
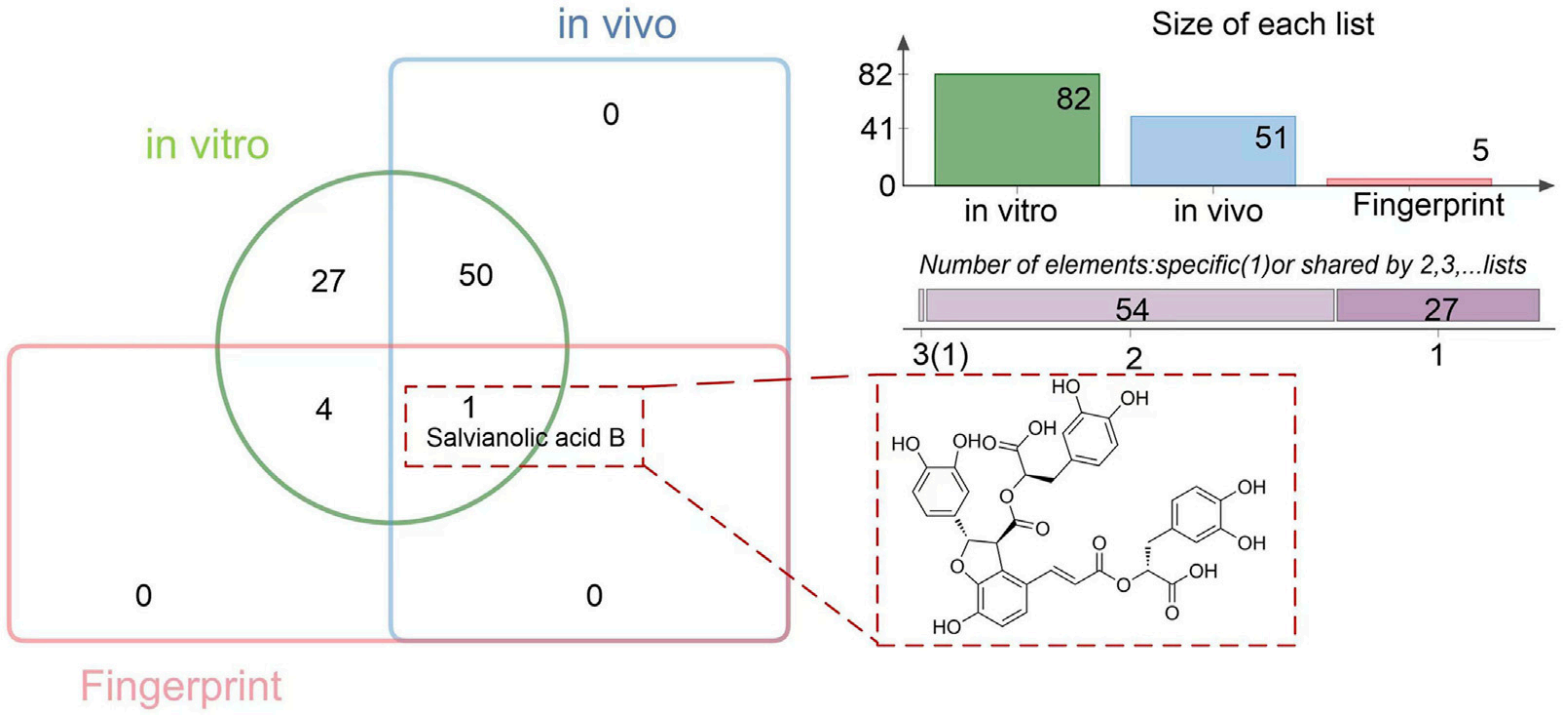
Quality marker prediction for TDC.

A total of 82 chemical constituents were identified from TDC extracts, and 51 prototype compounds were detected in rat plasma following TDC administration. Chemometric fingerprint analysis revealed five distinctive characteristic peaks. Comparative analysis using a Venn diagram indicated that Sal B may serve as a potential Q-marker for TDC (Figure 3).

### 3.4 Transcriptomic and pharmacological evaluation of sal B in HRD models

**FIGURE 4:**
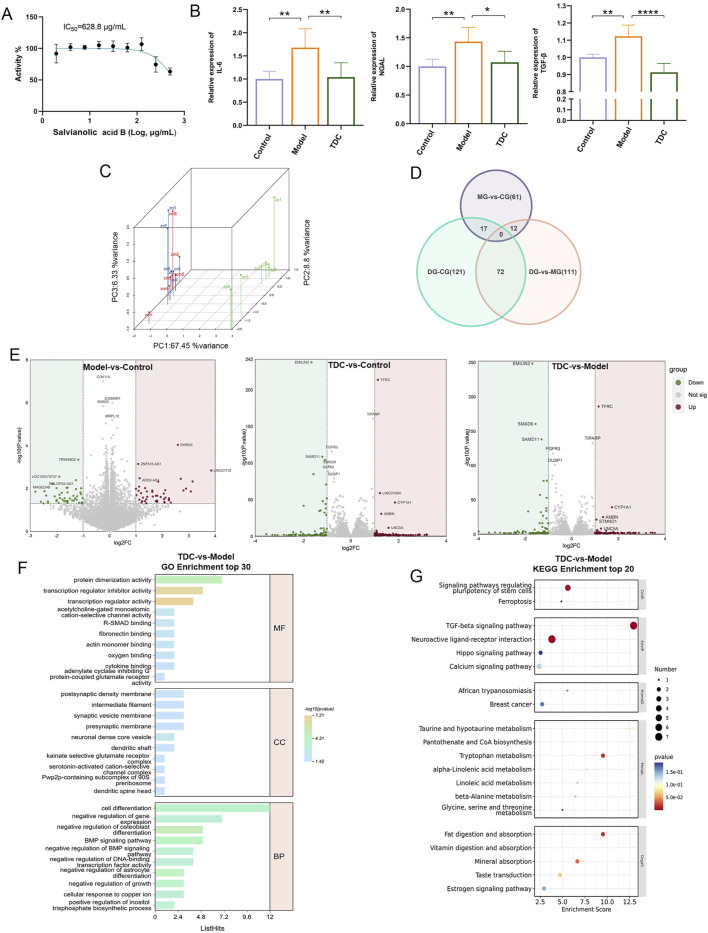
Pharmacological evaluation and transcriptomic analysis of Sal B in HRD. **(A)** CCK-8 assay revealed that Sal B inhibits cell viability. **(B)** Sal B treatment significantly reduced elevated IL-6, NGAL, and TGF-β levels in the model group. Data are depicted as mean ± SD; *p < 0.05, **p < 0.01, ***p < 0.001. **(C)** PCA analysis of the DEGs in each HEK293T cell group. **(D)** Venn diagram illustrating unique and overlapping DEGs among the different groups. **(E)** Upregulated and downregulated DEGs in the model vs. control, TDC vs. control, and TDC vs. model comparisons. **(F,G)** GO and KEGG analyses of DEGs, with the top 20 key pathways represented in bubble plots.

Cell viability was assessed using the CCK-8 assay (Figure 4A), which determined the half-maximal inhibitory concentration (IC_50_) of Sal B to be 628.8 μg/mL. To evaluate Sal B’s therapeutic efficacy against HRD, key biomarkers associated with inflammation and renal injury, including interleukin-6 (IL-6), neutrophil gelatinase-associated lipocalin (NGAL), and transforming growth factor-beta (TGF-β), were quantitatively measured. These markers were significantly elevated in the model group compared to the control (*p* < 0.01; Figure 4B), while Sal B treatment significantly decreased their levels (*p* < 0.05), suggesting its anti-inflammatory, anti-fibrotic, and nephroprotective potential.

Transcriptomic profiling of HEK293T cells following Sal B exposure revealed distinct expression changes. Principal component analysis (PCA) demonstrated that the first three components accounted for 67.45%, 8.8%, and 6.33% of the total variance, respectively (Figure 4C). Differentially expressed genes (DEGs) were identified using thresholds of |Log_2_ fold change| > 1 and p < 0.05 (Cao et al., 2023). Compared with the Control group, the Model group showed 90 significant DEGs (45 upregulated and 45 downregulated), while the TDC group showed 210 DEGs (120 upregulated and 90 downregulated). Between the TDC and Model groups, 195 DEGs were identified (104 upregulated and 91 downregulated) (Figures 4D,E).

To explore the potential biological mechanisms influenced by Sal B, Gene Ontology (GO) and Kyoto Encyclopedia of Genes and Genomes (KEGG) enrichment analyses were conducted on the DEGs (Chen et al., 2024; Wen et al., 2024). The top 20 enriched pathways were visualized using bubble plots (Figures 4F,G).

### 3.5 Transcriptomics combined with mendelian randomization to screen for biomarkers of sal B for HRD

**FIGURE 5:**
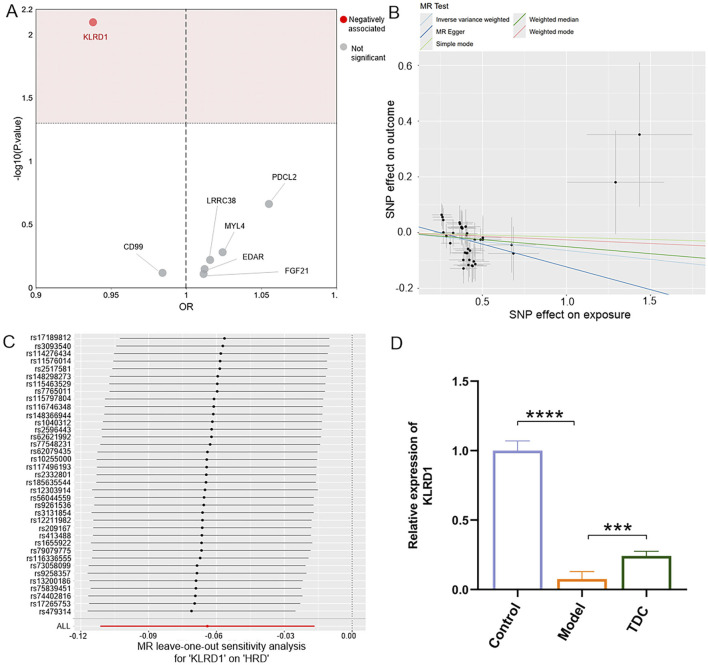
MR analysis and gene expression of KLRD1. **(A)** MR scatter plot showing a significant negative association of KLRD1 with HRD (highlighted in red). **(B)** MR regression plots of 5 MR methods (IVW, weighted mode, MR Egger, weighted median, and simple mode) indicated the effect of SNPs on exposure and outcome. **(C)** LOO sensitivity analysis for KLRD1 confirming robustness. **(D)** Relative KLRD1 mRNA expression in Control, Model, and TDC groups. Model group showed significantly reduced expression, partially restored after TDC treatment (****p < 0.0001; ***p < 0.001).

Using an absolute logFC >1 as the threshold, 396 DEGs were identified between the Model and Control groups, and 501 DEGs were found between the TDC and Model groups. Intersecting these datasets yielded 113 overlapping DEGs. Further comparison of these 113 DEGs with proteomic data comprising 2,925 proteins from the Finnish database identified seven shared targets. The MR analysis was then performed to assess the potential causal relationship between these candidates and HRD (Figure 5A).

Among the seven overlapping genes, only one demonstrated a statistically significant association (p < 0.05). Inverse-variance weighted (IVW) analysis indicated that Killer Cell Lectin Like Receptor D1 (KLRD1) serves as a protective factor against HRD (Figure 5B; Table 5). Furthermore, the MR-Egger intercept test (*p* > 0.05), Cochran’s Q test (*p* > 0.05), and MR-PRESSO analysis (*p* > 0.05) revealed no evidence of pleiotropy or heterogeneity (Table 6). Leave-one-out (LOO) sensitivity analysis confirmed that the MR estimates were not driven by any single SNP (Figure 5C). KLRD1 was significantly downregulated at the expression level in the HRD Model group compared to Controls (****p < 0.0001). Sal B treatment partially restored its expression (***p < 0.001) (Figure 5D).

**TABLE 5:** MR result of KLRD1 on HRD.

Exposure	Outcome	Method	SNP	*p*	OR (95% Cl)
KLRD1	HRD	MR Egger	37	0.09462	0.849 (0.705-1.023)
		Weighted median	37	0.15068	0.951 (0.888-1.018)
		Inverse variance weighted	37	0.00797	0.938 (0.895-0.983)
		Simple mode	37	0.81992	0.984 (0.855-1.132)
		Weighted mode	37	0.66438	0.974 (0.867-1.095)

**TABLE 6:** Sensitivity analyses for causality from KLRD1 on HRD.

Exposure	Outcome	Heterogeneity (IVW)		Horizontal pleiotropy (MR Egger)		MR-PRESSO
		Q	*p*	Egger-intercept	*p*	*p*
KLRD1	HRD	39.997	0.297	0.040	0.288	0.311

### 3.6 Untargeted metabolomics analysis identifies the differentially accumulated metabolites (DAMs)

**FIGURE 6:**
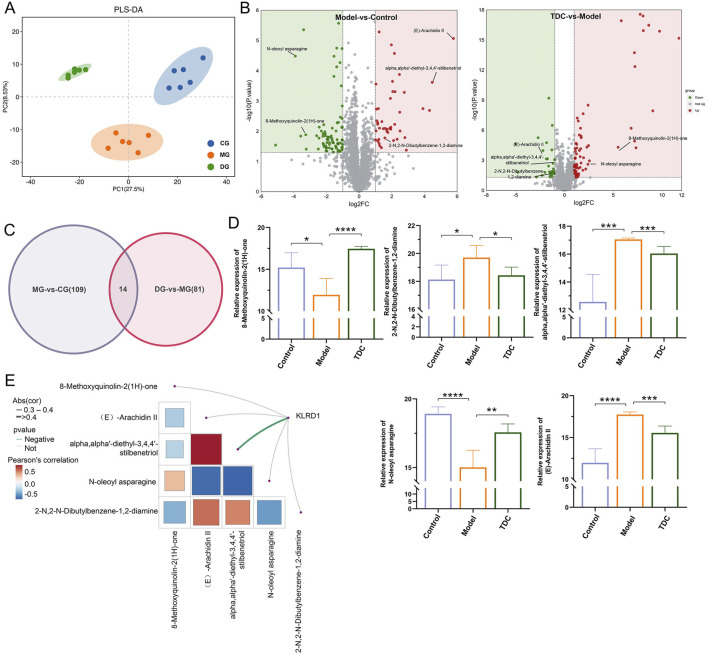
Metabolomic profiling and identification of KLRD1-associated differential metabolites. **(A)** PLS-DA plots reveal significant group differences and tight sample clustering. **(B)** Volcano plots highlighted the significantly altered metabolites in Model vs. Control (left) and TDC vs. Model (right) groups. Key metabolites are labeled. **(C)** The Venn diagram indicates the 14 overlapping metabolites between the two comparisons. **(D)** Quantitative analysis of five representative metabolites across groups; TDC treatment reversed abnormal changes in the model group (****p < 0.0001, ***p < 0.001, **p < 0.01, *p < 0.05) **(E)** Correlation analysis of KLRD1 and key metabolites. The color scale indicates Pearson’s correlation coefficient.

Partial Least Squares Discriminant Analysis (PLS-DA) was employed to evaluate the metabolomic profiles of the Control, Model, and TDC groups (Li J. et al., 2024). As illustrated in Figure 6A, the three groups displayed distinct metabolic signatures. Applying the criteria of absolute logFC >1, VIP >1, and *p* < 0.05 in the PLS-DA model (Teng and Wang, 2025; Xue et al., 2024), a total of 123 differentially accumulated metabolites (DAMs) were identified between the Control and Model groups 79 were downregulated and 44 were upregulated (Figure 6B). In comparison, 95 DAMs were detected between the TDC and Model groups, including 67 upregulated and 28 downregulated metabolites.

Furthermore, bioinformatics analysis was carried out to investigate the functional roles of these metabolites by constructing Venn diagrams (Figure 6C). The comparative analysis screened 5 DAMs as overlapping candidates for further functional characterization (Figure 6D). Correlation analysis between these 5 metabolites and key targets identified KLRD1 as a central molecule of interest. α,α′-diethyl-3,4,4′-stilbenetriol showed a significant negative correlation with KLRD1 expression (*p* < 0.05) (Figure 6E), suggesting that both may contribute critically to the therapeutic effects of Sal B in the context of HRD.

## 4 Discussion

To clarify the pharmacological basis and functional mechanisms of TDC in treating HRD, a comprehensive strategy integrating chemical profiling, chromatographic fingerprinting, transcriptomics, MR, and metabolomics was employed. A total of 82 chemical constituents were identified in TDC extracts using HPLC-Q-Exactive-MS, with organic acids, flavonoids, and quinones being the predominant classes. Among these, 51 prototype compounds were detected in rat plasma after oral administration, suggesting favorable bioavailability and potential *in vivo* activity. Fingerprint analysis across 10 independent TDC batches demonstrated high consistency, confirming the formulation’s chemical stability. Sal B emerged as a characteristic and recurrent peak in extract and plasma profiles and was designated a Q-marker through intersectional analysis. Sal B, a major water-soluble compound derived from Salvia miltiorrhiza, is recognized for its pronounced renoprotective properties. Recent studies have revealed that Sal B mitigates renal injury by inhibiting the Heparanase/Syndecan-1 (HPSE/SDC1) axis, reducing fibrosis-related markers, including Transforming Growth Factor Beta 1 (TGF-β1), Fibroblast Growth Factor 2 (FGF-2), and alpha-smooth muscle actin (α-SMA), and restoring epithelial integrity *in vitro* and *in vivo*. Furthermore, it acts synergistically with Tanshinone IIA to improve renal function parameters and attenuate inflammation by regulating the PI3K/Akt/NF-κB signaling pathway (Fu et al., 2024; Chen et al., 2022; Xu et al., 2024). In line with these findings, the present study demonstrated that Sal B treatment significantly lowered the elevated levels of IL-6, NGAL, and TGF-β in the HRD model, highlighting its anti-inflammatory, renoprotective, and anti-fibrotic properties. These results offer experimental support for the therapeutic potential of Sal B in managing HRD.

To investigate the molecular mechanisms underlying Sal B’s therapeutic effects against HRD, transcriptomic profiling was performed in HEK293T cells, identifying 210 DEGs. KEGG pathway enrichment analysis revealed that these DEGs were significantly associated with key biological pathways involved in inflammation, immune regulation, oxidative stress, signal transduction, and other processes highly relevant to HRD pathophysiology. These findings indicate that Sal B exerts broad transcriptional regulatory effects on disease-associated molecular networks. Two-sample MR analysis was conducted using GWAS data to assess causality further. The results identified KLRD1, which encodes a killer cell lectin-like receptor, as a protective factor against HRD. The robustness of this association was supported by the absence of horizontal pleiotropy and heterogeneity, as confirmed by MR-Egger regression, MR-PRESSO, and Cochran’s Q test. KLRD1 (CD94) plays a vital role in immune surveillance by regulating the activity of natural killer (NK) cells and specific T cell subsets. Dysregulation of KLRD1 has been implicated in developing various immune- and inflammation-related disorders. Reduced KLRD1 expression impairs NK cell function, potentially weakening innate immune defense and leading to abnormal renal inflammation. This dysregulation may contribute to hypertensive renal injury by amplifying inflammatory responses and promoting tissue damage (Li Q. et al. 2024; Wang C. M. et al., 2024; Wils et al., 2025). Since KLRD1 is associated with immune regulation, it may serve as a downstream effector of Sal B-induced immunomodulation. Its involvement implies that restoring NK cell-mediated immunological homeostasis via KLRD1 signaling may contribute to the therapeutic advantages of Sal B in hypertensive renal damage.

Untargeted metabolomics revealed five DAMs between the Sal B-treated and HRD groups, suggesting a significant metabolic reprogramming associated with Sal B intervention. Especially, α,α′-diethyl-3,4,4′-stilbenetriol showed a strong negative correlation with KLRD1 expression, pointing to a potential immunometabolic regulatory axis modulated by Sal B. The α,α′-Diethyl-3,4,4′-stilbenetriol, a stilbene derivative, shows diverse biological activities, including antioxidant, anti-inflammatory, and immunomodulatory effects. However, similar to other stilbenes such as resveratrol, it may carry a risk of renal toxicity. Resveratrol has been shown to exert dose-dependent nephrotoxic effects, with higher concentrations leading to kidney injury and aggravation of renal fibrosis (Shaito et al., 2020). The present study indicates that α,α′-Diethyl-3,4,4′-stilbenetriol may act as a downstream mediator that bridges metabolic alterations with immune responses in the context of hypertensive renal pathology. These integrated transcriptomic and metabolomic findings support the role of Sal B, identified as a Q-marker of TDC, in improving TDC’s therapeutic effects against HRD, likely by orchestrating the regulation of immune and metabolic pathways. This study has several limitations. First, although MR offers strong causal inference, its reliability depends heavily on the quality and comprehensiveness of the underlying GWAS datasets. Limitations in these datasets may affect the accuracy and generalizability of the results. Therefore, further research is needed to functionally validate the roles of KLRD1 and its associated metabolites in the pathogenesis and treatment of HRD. Such studies should aim to elucidate the molecular mechanisms and interactions involved, which would provide more definitive evidence of their therapeutic relevance. Second, while our findings are encouraging, future studies should also address the quantification and pharmacokinetics of key plasma constituents, including α,α′-Diethyl-3,4,4′-stilbenetriol. A detailed understanding of this compound’s absorption, distribution, metabolism, and excretion (ADME) profiles will be critical for developing an effective and clinically applicable dosing strategy.

## 5 Conclusion

In conclusion, this study combined chemical fingerprinting, transcriptomic analysis, and Mendelian randomization to identify Sal B and KLRD1 as pivotal quality and therapeutic efficacy markers. Furthermore, it uncovered novel metabolic regulators potentially implicated in the pathogenesis of HRD. Together, these results offer a comprehensive, multi-layered understanding of TDC’s pharmacological profile, underscoring Sal B as a bioactive compound with dual significance for therapeutic action and quality assurance.

## Data Availability

The data presented in the study are deposited in the NCBI Sequence Read Archive (SRA) database, accession number PRJNA1297347.
