# Supplementary material for: Association analysis-based screening strategy for quality markers of Tengdan capsule in the treatment of hypertensive renal disease
Source: Front Pharmacol. 2025 Aug 4;16:1647921. doi: 10.3389/fphar.2025.1647921 (PMC12358744; doi:10.3389/fphar.2025.1647921)
Supplement: Supplementary file 1 [file Table1.docx]

Table S1 Constituents in TDC Extracts

| NO. | Identification | t_R_(min) | MF | Product ion | Mass | Mass (Tgt) | Diff (ppm) | Fragment Ions（m/z） | Classification |
| --- | --- | --- | --- | --- | --- | --- | --- | --- | --- |
| 1 | Arginine | 1.03 | C_6_H_14_O_2_N_4_ | [M-H]^-^ | 173.10330 | 173.10164 | -9.602 | 173.10164,  156.07504,  114.05325 | Amino acids |
| 2 | Quinic acid | 1.17 | C_7_H_12_O_6_ | [M-H]^-^ | 191.05501 | 191.05344 | -8.241 | 191.05344,  173.00630,  127.03751,  101.02207 | Organic acids |
| 3 | Methyl dihydrotanshinate | 1.49 | C_20_H_20_O_5_ | [M+H]+ | 341.13835 | 341.13596 | 7.007 | 341.13596,  281.16321,  263.10437,  207.11372 | Organic acids |
| 4 | Citric acid | 1.88 | C_6_H_8_O_7_ | [M-H]^-^ | 191.01862 | 191.01703 | -8.371 | 191.01703,  173.00624,  111.00625,  87.00639 | Organic acids |
| 5 | Adenosine | 2.90 | C_10_H_13_N_5_O_4_ | [M＋H]^+^ | 268.10403 | 268.10550 | 5.481 | 268.10550,  136.06285 | Others |
| 6 | Gallic acid | 3.20 | C_7_H_6_O_5_ | [M-H]^-^ | 169.01314 | 169.01146 | -9.998 | 169.01146,  125.02190,  97.02718 | Organic acids |
| 7 | Salvianic acid A | 4.62 | C_9_H_10_O_5_ | [M+H]+ | 199.06009 | 199.06046 | 1.809 | 199.06046,  181.09837,  137.07161,  125.06155 | Organic acids |
| 8 | Sorbitol | 7.22 | C_6_H_14_O_6_ | [M-H]^-^ | 181.07066 | 181.06908 | -8.751 | 181.06908,  119.03242,  109.02676,  89.02203 | Others |
| 9 | 5-Hydroxymethylfurfural | 7.42 | C_6_H_6_O_3_ | [M+H]+ | 127.03897 | 127.04002 | 8.260 | 127.04002,  109.02946 | Others |
| 10 | Neochlorogenic acid | 8.02 | C_16_H_18_O_9_ | [M+H]+ | 355.10235 | 355.10168 | -1.911 | 355.10168,  135.04514 | Organic acids |
| 11 | Dihydrocaffeic acid | 10.66 | C_9_H_10_O_4_ | [M-H]- | 181.04953 | 181.04811 | -7.872 | 181.04811,  163.03723 | Organic acids |
| 12 | Chlorogenic acid | 10.69 | C_16_H_18_O_9_ | [M-H]^-^ | 353.08670 | 353.08511 | -4.527 | 353.08511,  191.05345,  179.03232,  173.04250,  135.04260,  85.02711 | Organic acids |
| 13 | Geniposidic acid* | 10.81 | C_16_H_22_O_10_\ | [M-H]- | 373.11292 | 373.11191 | -1.322 | 373.11243,  211.05858,  193.04788,  123.04264 | Glucosides |
| 14 | Shikimic acid | 12.49 | C_7_H_12_O_5_ | [M-H]^-^ | 175.06009 | 175.05849 | -9.197 | 175.05849,  113.05833 | Organic acids |
| 15 | Tanshinic acid methyl ester | 13.25 | C_10_H_12_O_5_ | [M-H]^-^ | 211.06009 | 211.05853 | -7.438 | 211.05853,  165.05266 | Others |
| 16 | Vanillic acid | 13.51 | C_8_H_8_O_4_ | [M-H]^-^ | 167.03388 | 167.03224 | -9.805 | 167.03224,  152.00891,  121.02702,  123.04266 | Organic acids |
| 17 | Caffeic acid | 14.19 | C_9_H_8_O_4_ | [M-H]^-^ | 179.03388 | 179.03226 | -9.078 | 179.03226,  136.04594,  135.04259,  107.04784,  89.02210 | Organic acids |
| 18 | Phenylalanine | 14.44 | C_9_H_11_NO_2_ | [M+H]+ | 166.08625 | 166.08730 | 6.291 | 166.08730,  120.08173,  93.07104 | Amino acids |
| 19 | Salvianolic acid F | 14.48 | C_17_H_14_O_6_ | [M-H]^-^ | 313.07066 | 313.06894 | -5.509 | 313.06894,  269.07950,  159.04207 | Organic acids |
| 20 | Mitragynine | 16.50 | C_21_H_24_N_2_O_4_ | [M+H]+ | 369.18088 | 369.18280 | 5.191 | 369.18280 | Alkaloids |
| 21 | Ferulic Acid * | 16.59 | C_10_H_10_O_4_ | [M-H]^-^ | 193.04953 | 193.04799 | -8.005 | 193.04799,  192.06210,  191.05336,  178.02431,  149.02184,  134.03508 | Organic acids |
| 22 | Vanillin | 17.49 | C_8_H_8_O_3_ | [M-H]^-^ | 151.03897 | 151.03752 | -9.604 | 151.03752,  136.01410 | Others |
| 23 | Plantainoside A | 17.56 | C_11_H_19_N_3_O_2_ | [M-H]^-^ | 224.13935 | 224.13789 | -6.529 | 224.13789,  141.08958 | Flavonoids |
| 24 | Tetramethylpyrazine | 18.08 | C_8_H_12_N_2_ | [M+H]+ | 137.10732 | 137.10832 | 7.257 | 137.10832,  122.08490 | Alkaloids |
| 25 | p-Coumaric acid | 18.20 | C_9_H_8_O_3_ | [M+H]+ | 165.05462 | 165.05566 | 6.297 | 165.05566,  147.04520,  119.01995 | Organic acids |
| 26 | Corynoxeine | 19.00 | C_22_H_26_N_2_O_4_ | [M+H]+ | 383.19653 | 383.19152 | 5.183 | 383.19152,  160.07655 | Alkaloids |
| 27 | D-Tetrandrine | 19.15 | C_38_H_42_N_2_O_6_ | [M+H]+ | 623.31156 | 623.31488 | 5.321 | 623.31488,  592.27258,  395.19763,  190.08772 | Alkaloids |
| 28 | Hyperoside * | 19.66 | C_21_H_20_O_12_ | [M-H]^-^ | 463.08710 | 463.08435 | -5.944 | 463.08435,  300.02484,  255.02739,  151.00108 | Flavonoids |
| 29 | Rutin * | 19.79 | C_27_H_30_O_16_ | [M-H]^-^ | 609.14501 | 609.14197 | -4.992 | 609.14197,  300.02478,  271.02206 | Flavonoids |
| 30 | Chuanxiong phenol | 19.97 | C_12_H_14_O_3_ | [M+H]+ | 207.10157 | 207.10289 | 6.370 | 207.10289,  189.09254,  163.11281,  121.06595,  93.07110 | Organic acids |
| 31 | Coumarin | 20.21 | C_9_H_6_O_2_ | [M-H]^-^ | 145.02840 | 145.02715 | -8.660 | 145.02715,  115.03753，  85.02718 | Others |
| 32 | Salvianolic acid B | 20.60 | C36H30O16 | [M+H]^+^ | 719.16066 | 719.16248 | 2.529 | 719.16248 | Organic acids |
| 33 | Salicylic acid | 20.83 | C_7_H_6_O_3_ | [M-H]- | 137.02332 | 137.02196 | -9.929 | 137.02196，  93.03233 | Organic acids |
| 34 | Przewalskic acid A | 20.85 | C_18_H_14_O_8_ | [M-H]^-^ | 357.06040 | 357.05859 | -5.332 | 357.05859,  339.04804,  311.05344 | Organic acids |
| 35 | Quercetin | 20.96 | C_15_H_10_O_7_ | [M+H]+ | 303.04992 | 303.05176 | 6.041 | 303.05176 | Flavonoids |
| 36 | Taxifolin | 21.21 | C_15_H_12_O_7_ | [M-H]^-^ | 303.04992 | 303.04849 | -4.749 | 303.04849,  257.04315 | Flavonoids |
| 37 | Pulegone | 21.61 | C_10_H_16_O | [M+H]+ | 153.12739 | 153.12825 | 5.605 | 153.12825,  107.08657,  93.07099,  69.07099 | Terpenes |
| 38 | Rhamnocitri | 21.66 | C_16_H_12_O_6_ | [M-H]^-^ | 299.05501 | 299.05350 | 5.064 | 299.05350 | Flavonoids |
| 39 | Calycosin-7-o-beta-d-glucoside | 21.73 | C22H22O10 | [M-H]^-^ | 445.11292 | 445.11136 | -3.512 | 445.11136 | Glucosides |
| 40 | Afzelin | 22.29 | C_21_H_20_O_10_ | [M-H]^-^ | 431.09727 | 431.09540 | -4.345 | 431.09540,  285.03751,  255.02698 | Flavonoids |
| 41 | Calycosin | 22.69 | C_16_H_12_O_5_ | [M-H]^-^ | 283.06009 | 283.05859 | -5.334 | 283.05859,  268.03522,  211.03700,  148.01511,  135.00629 | Organic acids |
| 42 | Catechin | 22.50 | C_15_H_14_O_6_ | [M+H]+ | 291.08631 | 291.08694 | 2.148 | 291.08694,  139.07855,  123.04706 | Flavonoids |
| 43 | Naringenin chalcone | 22.93 | C_15_H_12_O_5_ | [M+H]+ | 273.07575 | 273.07712 | 5.017 | 273.07712,  189.05602 | Flavonoids |
| 44 | Luteolin * | 23.03 | C_15_H_10_O_6_ | [M-H]^-^ | 285.03936 | 285.03793 | -5.033 | 285.03793 | Flavonoids |
| 45 | Senkyunolide H | 23.09 | C_12_H_16_O_4_ | [M+H]+ | 225.11213 | 225.11327 | 5.040 | 225.11327,  207.10252,  189.12897,  179.07173,  165.09193,  161.09697,  151.07648,  147.11797,  119.08654,  105.07108 | Coumarins |
| 46 | Malic acid | 23.17 | C_4_H_6_O_5_ | [M-H]^-^ | 133.01314 | 133.01221 | -7.065 | 133.01221,  115.00117 | Organic acids |
| 47 | Resveratrol | 23.23 | C_14_H_12_O_3_ | [M+H]+ | 229.08592 | 229.08696 | 4.536 | 229.08696 | Organic acids |
| 48 | Salvianolicacid C | 23.43 | C_26_H_20_O_10_ | [M+H]+ | 493.11292 | 493.11465 | 3.502 | 493.11465,  313.07251，  295.06183,  199.14899 | Organic acids |
| 49 | Apigenin | 23.55 | C_15_H_10_O_5_ | [M-H]^-^ | 269.04444 | 269.04303 | -5.277 | 269.04303,  225.14693,  65.00215 | Flavonoids |
| 50 | Naringenin | 24.09 | C_15_H_12_O_5_ | [M-H]^-^ | 271.06009 | 271.05896 | -4.205 | 271.05896,  253.14119,  151.00261 | Flavonoids |
| 51 | p-Coumaric acid | 24.36 | C_9_H_8_O_3_ | [M-H]- | 163.03897 | 163.03740 | -9.633 | 163.03740,  119.04791,  93.03252 | Organic acids |
| 52 | Tanshindiol  C | 24.91 | C_18_H_16_O_5_ | [M+H]+ | 313.10705 | 313.10934 | 7.313 | 313.10934,  267.13889,  249.12665 | Organic acids |
| 53 | Methyl salvianolate | 24.94 | C_20_H_18_O_5_ | [M+H]+ | 339.12270 | 339.12131 | -4.009 | 339.12131,  279.10281,  261.15082 | Organic acids |
| 54 | Oleanolic acid | 25.02 | C_30_H_48_O_3_ | [M+H]+ | 457.36762 | 457.36932 | 3.713 | 457.36932,  175.14893,  133.08705 | Terpenes |
| 55 | Formononetin | 25.71 | C_16_H_12_O_4_ | [M-H]^-^ | 267.06518 | 267.06375 | -5.374 | 267.06375,  252.04016,  195.04179,  132.02003 | Flavonoids |
| 56 | Senkyunolide A | 25.74 | C_12_H_16_O_2_ | [M+H]+ | 193.12230 | 193.12376 | 7.572 | 193.12376,  175.11301,  147.11786,  137.06081,  119.08647 | Coumarins |
| 57 | Hydrogenated dihydroxytanshinone  ⅡA | 26.97 | C_19_H_20_O_5_ | [M+H]+ | 329.13835 | 329.13922 | 2.643 | 329.13922,  311.12939,  267.13947 | Quinone |
| 58 | Trijuganone C | 27.60 | C_20_H_20_O_5_ | [M+H]+ | 341.13835 | 341.13989 | 4.514 | 341.13989 | Quinone |
| 59 | Tanshinaldehyde | 27.83 | C_19_H_16_O_4_ | [M+H]+ | 309.11213 | 309.11380 | 5.385 | 309.11380,  265.12390 | Others |
| 60 | Dibutyl phthalate | 27.93 | C_16_H_22_O_4_ | [M+H]+ | 279.15908 | 279.16122 | 7.645 | 279.16122,  149.02423,  121.10288 | Organic acids |
| 61 | Trijuganone A | 28.13 | C_18_H_14_O_4_ | [M+H]+ | 295.09648 | 295.09833 | 6.251 | 295.09833,  277.08765,  263.08189,  249.09241 | Quinone |
| 62 | Neotanshinquinone D | 28.46 | C_21_H_20_O_4_ | [M+H]+ | 337.14343 | 337.14258 | -2.538 | 337.14258 | Quinone |
| 63 | Tanshinone  V | 29.06 | C_19_H_22_O_4_ | [M+H]+ | 315.15908 | 315.16119 | 6.677 | 315.16119,  297.18643,  269.15552 | Quinone |
| 64 | Ginsenoside Rd | 29.54 | C_48_H_82_O_18_ | [M-H]^-^ | 945.54174 | 945.54456 | 2.980 | 945.54456,  783.48871 | Glucosides |
| 65 | Neocryptotanshinone | 30.81 | C_19_H_22_O_4_ | [M-H]^-^ | 313.14343 | 313.14200 | -4.585 | 313.14200 | Quinone |
| 66 | Tanshinone I | 31.13 | C_18_H_12_O_3_ | [M+H]+ | 277.08592 | 277.08768 | 6.349 | 277.08768,  249.09145 | Quinone |
| 67 | Dihydrotanshinone Ⅰ | 31.16 | C_18_H_14_O_3_ | [M-H]^-^ | 277.08592 | 277.08459 | -4.803 | 277.08459 | Quinone |
| 68 | Tormentic acid | 31.44 | C_30_H_48_O_5_ | [M-H]^-^ | 487.3418 | 487.33951 | -4.701 | 487.33951 | Organic acids |
| 69 | Deoxocarnosol | 31.48 | C_20_H_28_O_3_ | [M-H]^-^ | 315.19547 | 315.19388 | -5.048 | 315.19388 | Organic acids |
| 70 | Sugiol | 31.66 | C_20_H_28_O_2_ | [M-H]^-^ | 299.20055 | 299.19897 | -5.303 | 299.19897 | Organic acids |
| 71 | Dehydroneotanshinone | 31.76 | C_19_H_20_O_2_ | [M+H]+ | 281.15360 | 281.15555 | 6.913 | 281.15555 | Quinone |
| 72 | Neotanshinquinone B | 32.18 | C_18_H_16_O_3_ | [M+H]+ | 281.11722 | 281.11914 | 6.827 | 281.11914,  235.11340 | Quinone |
| 73 | α-Hydroxylinoleic acid | 32.38 | C_18_H_32_O_3_ | [M-H]^-^ | 295.22677 | 295.22528 | -5.051 | 295.22528,  277.21481 | Organic acids |
| 74 | Neotanshinone | 32.66 | C_19_H_22_O_2_ | [M+H]+ | 283.16925 | 283.17120 | 6.863 | 283.17120,  265.16031 | Quinone |
| 75 | Tanshinphenolquinone Ⅱ | 32.67 | C_19_H_20_O_4_ | [M-H]^-^ | 311.12778 | 311.12628 | -4.839 | 311.12628,  293.20981,  275.20081,  247.20576 | Quinone |
| 76 | Isocryptotanshinone | 32.78 | C_19_H_20_O_3_ | [M-H]^-^ | 295.13287 | 295.13226 | -2.070 | 295.13226,  277.21487 | Quinone |
| 77 | Tanshinone IIA | 32.83 | C_19_H_18_O_3_ | [M+H]+ | 295.13287 | 295.13492 | 6.943 | 295.13492 | Quinone |
| 78 | Linolenic acid | 35.28 | C_18_H_30_O_2_ | [M-H]^-^ | 277.21620 | 277.21478 | -5.146 | 277.21478 | Organic acids |
| 79 | Astragaloside II | 35.56 | C_35_H_58_O_9_ | [M-H]^-^ | 621.39970 | 621.39648 | -5.198 | 621.39648 | Glucosides |
| 80 | Linoleic acid | 36.52 | C_18_H_32_O_2_ | [M-H]^-^ | 279.23185 | 279.23044 | -5.074 | 279.23044,  261.22034 | Organic acids |
| 81 | Palmitic acid | 37.76 | C_16_H_32_O_2_ | [M-H]^-^ | 255.23185 | 255.23055 | -5.120 | 255.23055 | Organic acids |
| 82 | Oleic acid | 38.44 | C_18_H_34_O_2_ | [M-H]^-^ | 281.24750 | 281.24612 | -4.931 | 281.24612 | Organic acids |

Table S2 Absorbed Prototype Compounds in Plasma in TDC

| NO. | Identification | tR(min) | MF | Product ion | Mass | Mass (Tgt) | Diff (ppm) | Fragment Ions（m/z） | Classification |
| --- | --- | --- | --- | --- | --- | --- | --- | --- | --- |
| 1 | Shikimic acid | 1.32 | C_7_H_12_O_5_ | [M-H]^-^ | 175.06009 | 175.06114 | 5.941 | 175.06114,  113.02371 | Organic acids |
| 2 | Quinic acid | 1.53 | C_7_H_12_O_6_ | [M-H]^-^ | 191.05501 | 191.05641 | 7.304 | 191.05641,  173.00885 | Organic acids |
| 3 | Malic acid | 1.60 | C_4_H_6_O_5_ | [M-H]^-^ | 133.01314 | 133.01373 | 4.362 | 133.01373,  115.00287 | Organic acids |
| 4 | Citric acid | 1.95 | C_6_H_8_O_7_ | [M-H]^-^ | 191.01862 | 191.01987 | 6.497 | 191.01987,  173.00885,  111.00795,  87.00773 | Organic acids |
| 5 | p-Coumaric acid | 2.37 | C_9_H_8_O_3_ | [M-H]^+^ | 165.05462 | 165.05464 | 0.117 | 165.05464,  147.04413,  119.04950 | Organic acids |
| 6 | Salvianic acid A | 2.76 | C_9_H_10_O_5_ | [M-H]^+^ | 199.06009 | 199.05965 | -2.260 | 199.05965,  181.09734,  137.05997,  125.09652 | Organic acids |
| 7 | Tetramethylpyrazine | 3.68 | C_8_H_12_N_2_ | [M-H]^+^ | 137.10732 | 137.10747 | 1.058 | 137.10747,  122.06032 | Alkaloids |
| 8 | 5-Hydroxymethylfurfural | 6.57 | C_6_H_6_O_3_ | [M-H]^+^ | 127.03897 | 127.03932 | 2.750 | 127.03932,  109.02894， | Others |
| 9 | Gallic acid | 10.31 | C_7_H_6_O_5_ | [M-H]^-^ | 169.01314 | 169.01401 | 5.090 | 169.01401，125.02377,  97.02898 | Organic acids |
| 10 | Dihydrocaffeic acid | 11.01 | C_9_H_10_O_4_ | [M-H]- | 181.04953 | 181.04994 | 2.235 | 181.04994,  163.03961 | Organic acids |
| 11 | Vanillic acid | 13.16 | C_8_H_8_O_4_ | [M-H]^-^ | 167.03388 | 167.03465 | 4.579 | 167.03465,  121.02869,  123.04447 | Organic acids |
| 12 | Tanshinic acid methyl ester | 13.19 | C_10_H_12_O_5_ | [M-H]^-^ | 211.06009 | 211.06166 | 7.392 | 211.06166,  165.05545 | Others |
| 13 | Dehydroneotanshinone | 14.52 | C_19_H_20_O_2_ | [M-H]^+^ | 281.15360 | 281.15161 | -7.101 | 281.15161 | Quinone |
| 14 | Isocryptotanshinone | 15.81 | C_19_H_20_O_3_ | [M-H]^-^ | 295.13287 | 295.13153 | -4.544 | 295.13153,  277.21487， | Quinone |
| 15 | Mitragynine | 15.93 | C_21_H_24_N_2_O_4_ | [M-H]^+^ | 369.18088 | 369.18149 | 1.642 | 369.18149 | Alkaloids |
| 16 | Neotanshinone | 16.11 | C_19_H_22_O_2_ | [M-H]^+^ | 283.16925 | 283.16809 | -4.119 | 283.16809,  265.07611 | Quinone |
| 17 | Caffeic acid | 17.04 | C_9_H_8_O_4_ | [M-H]^-^ | 179.03388 | 179.03525 | 7.623 | 179.03525,  136.07629,  135.04475,  107.04956,  89.02348 | Organic acids |
| 18 | Plantainoside A | 17.83 | C_11_H_19_N_3_O_2_ | [M-H]^-^ | 224.13935 | 224.14111 | 7.837 | 224.14111,  141.09171 | Flavonoids |
| 19 | Salicylic acid | 18.50 | C_7_H_6_O_3_ | [M-H]- | 137.02332 | 137.02391 | 4.302 | 137.02391，93.03365 | Organic acids |
| 20 | Corynoxeine | 18.86 | C_22_H_26_N_2_O_4_ | [M-H]^+^ | 383.19653 | 383.19705 | 1.347 | 383.19705,  160.07570 | Alkaloids |
| 21 | Chuanxiong phenol | 20.06 | C_12_H_14_O_3_ | [M-H]^+^ | 207.10157 | 207.10173 | 0.768 | 207.10173,  189.09145,  163.11150,  121.06535,  93.07050 | Organic acids |
| 22 | Salvianolic acid B | 20.60 | C36H30O16 | [M+H]^+^ | 719.16066 | 719.16241 | 2.432 | 719.16241 | Organic acids |
| 23 | Pulegone | 20.73 | C_10_H_16_O | [M-H]^+^ | 153.12739 | 153.12737 | -0.142 | 153.12737,  107.08592,  93.07045,  69.07065 | Terpenes |
| 24 | Senkyunolide H | 21.78 | C_12_H_16_O_4_ | [M-H]^+^ | 225.11213 | 225.11229 | 0.686 | 225.11229,  207.10161,  189.09119,  179.10703,  165.09090,  161.09573,  151.07510,  147.11684,  119.08577,  105.03393 | Coumarins |
| 25 | Vanillin | 22.60 | C_8_H_8_O_3_ | [M-H]^-^ | 151.03897 | 151.03969 | 4.763 | 151.03969,  136.01602 | Others |
| 26 | Resveratrol | 23.27 | C_14_H_12_O_3_ | [M-H]^+^ | 229.08592 | 229.08606 | 0.608 | 229.08606 | Organic acids |
| 27 | Deoxocarnosol | 24.29 | C_20_H_28_O_3_ | [M-H]^-^ | 315.19547 | 315.19812 | 8.404 | 315.19812， | Organic acids |
| 28 | Hydrogenated dihydroxytanshinone  ⅡA | 24.53 | C_19_H_20_O_5_ | [M-H]^+^ | 329.13835 | 329.13812 | -0.699 | 329.13812,  311.12753,  267.13806 | Quinone |
| 29 | Methyl salvianolate | 25.03 | C_20_H_18_O_5_ | [M-H]^+^ | 339.12270 | 339.12070 | -5.898 | 339.12131,  279.10281,  261.15082 | Organic acids |
| 30 | Taxifolin | 25.24 | C_15_H_12_O_7_ | [M-H]^-^ | 303.04992 | 303.05222 | 7.559 | 303.05222,  257.15361 | Flavonoids |
| 31 | Senkyunolide A | 25.37 | C_12_H_16_O_2_ | [M-H]^+^ | 193.12230 | 193.12270 | 2.039 | 193.12270，147.11684 | Coumarins |
| 32 | Trijuganone A | 25.53 | C_18_H_14_O_4_ | [M-H]^+^ | 295.09648 | 295.09534 | -3.882 | 295.09534,  277.04953,  263.23727, 249.14839 | Quinone |
| 33 | Formononetin | 25.70 | C_16_H_12_O_4_ | [M-H]^-^ | 267.06518 | 267.06757 | 8.929 | 267.06757,  252.04381,  195.13884 | Flavonoids |
| 34 | Przewalskic acid A | 26.04 | C_18_H_14_O_8_ | [M-H]^-^ | 357.06040 | 357.06387 | 9.455 | 357.06387 | Organic acids |
| 35 | Sorbitol | 26.29 | C_6_H_14_O_6_ | [M-H]^-^ | 181.07066 | 181.07214 | 8.148 | 181.07214,  119.03438,  89.02344 | Others |
| 36 | Methyl dihydrotanshinate | 27.69 | C_20_H_20_O_5_ | [M-H]^+^ | 341.13835 | 341.13818 | -0.499 | 341.13818,  263.10693,  207.10181 | Organic acids |
| 37 | Tanshinaldehyde | 27.93 | C_19_H_16_O_4_ | [M-H]^+^ | 309.11213 | 309.11249 | 1.147 | 309.11249,  265.12265 | Others |
| 38 | Dibutyl phthalate | 27.94 | C_16_H_22_O_4_ | [M-H]^+^ | 279.15908 | 279.15948 | 1.412 | 279.15948,  149.02353, | Organic acids |
| 39 | Salvianolic acid F | 28.18 | C_17_H_14_O_6_ | [M-H]^-^ | 313.07066 | 313.07303 | 7.555 | 313.07303,  269.15659,  158.38579 | Organic acids |
| 40 | Neocryptotanshinone | 28.46 | C_19_H_22_O_4_ | [M-H]^-^ | 313.14343 | 313.14651 | 9.818 | 313.14651 | Quinone |
| 41 | Neotanshinquinone D | 28.56 | C_21_H_20_O_4_ | [M-H]^+^ | 337.14343 | 337.14124 | -6.512 | 337.14258 | Quinone |
| 42 | Tanshinphenolquinone Ⅱ | 29.87 | C_19_H_20_O_4_ | [M-H]^-^ | 311.12778 | 311.13065 | 9.207 | 311.13065,  293.21359,  275.20328,  247.20894， | Quinone |
| 43 | Neotanshinquinone B | 29.99 | C_18_H_16_O_3_ | [M-H]^+^ | 281.11722 | 281.11749 | 0.957 | 281.11749,  235.11182 | Quinone |
| 44 | Dihydrotanshinone Ⅰ | 30.08 | C_18_H_14_O_3_ | [M-H]^-^ | 277.08592 | 277.08514 | -2.818 | 277.08514 | Quinone |
| 45 | Tanshinone IIA | 30.06 | C_19_H_18_O_3_ | [M-H]^+^ | 295.13287 | 295.13361 | 2.504 | 295.13361 | Quinone |
| 46 | Oleanolic acid | 30.62 | C_30_H_48_O_3_ | [M-H]^+^ | 457.36762 | 457.37082 | 6.992 | 457.37082,  175.14880,  133.10188 | Terpenes |
| 47 | Tanshinone  V | 30.90 | C_19_H_22_O_4_ | [M-H]^+^ | 315.15908 | 315.15903 | -0.177 | 315.15903,  297.14890,  269.15381 | Quinone |
| 48 | Tanshindiol  C | 31.09 | C_18_H_16_O_5_ | [M-H]^+^ | 313.10705 | 313.10791 | 2.746 | 313.1079,  267.13815 | Organic acids |
| 49 | Sugiol | 32.10 | C_20_H_28_O_2_ | [M-H]^-^ | 299.20055 | 299.20084 | 0.947 | 299.20084 | Organic acids |
| 50 | α-Hydroxylinoleic acid | 32.42 | C_18_H_32_O_3_ | [M-H]^-^ | 295.22677 | 295.22931 | 8.599 | 295.22931,  277.21884 | Organic acids |
| 51 | Tormentic acid | 36.96 | C_30_H_48_O_5_ | [M-H]^-^ | 487.3418 | 487.34665 | 9.950 | 487.34665 | Organic acids |
